# Developing an Environmental Health Sciences COVID-19 Research Agenda: Results from the NIEHS Disaster Research Response (DR2) Work Group’s Modified Delphi Method

**DOI:** 10.3390/ijerph17186842

**Published:** 2020-09-19

**Authors:** Nicole A. Errett, Marilyn Howarth, Kimberley Shoaf, Megan Couture, Steven Ramsey, Richard Rosselli, Sara Webb, April Bennett, Aubrey Miller

**Affiliations:** 1 Department of Environmental and Occupational Health Sciences, University of Washington School of Public Health, Seattle, WA 98195, USA; 2 Center of Excellence in Environmental Toxicology, Perelman School of Medicine, University of Pennsylvania, Philadelphia, PA 19104, USA; howarthmv@gmail.com; 3 Division of Public Health, University of Utah, Salt Lake City, UT 84108, USA; kimberley.shoaf@utah.edu; 4 Social & Scientific Systems, Inc., Durham, NC 27703, USA; megan.t.couture@gmail.com (M.C.); stkeram@gmail.com (S.R.); richard.rosselli@gmail.com (R.R.); sara.webb@dlhcorp.com (S.W.); 5 Contractor, National Institute of Environmental Health Sciences; Bethesda, MD 20892, USA; april.bennett@nih.gov; 6 National Institute of Environmental Health Sciences; Bethesda, MD 20892, USA; miller.aubrey@nih.gov

**Keywords:** environmental health, COVID-19, research priorities

## Abstract

Leveraging the community of practice recently established through the U.S. National Institute of Environmental Health Sciences (NIEHS) Disaster Research Response (DR2) working group, we used a modified Delphi method to identify and prioritize environmental health sciences Severe Acute Respiratory Syndrome Coronavirus 2 (SARS-CoV-2) and associated Coronavirus Disease 2019 (COVID-19) research questions. Twenty-six individuals with broad expertise across a variety of environmental health sciences subdisciplines were selected to participate among 45 self-nominees. In Round 1, panelists submitted research questions and brief justifications. In Round 2, panelists rated the priority of each question on a nine-point Likert scale. Responses were trichotomized into priority categories (low priority; medium priority; and high priority). A research question was determined to meet consensus if at least 69.2% of panelists rated it within the same priority category. Research needs that did not meet consensus in round 2 were redistributed for re-rating. Fourteen questions met consensus as high priority in round 2, and an additional 14 questions met consensus as high priority in round 3. We discuss the impact and limitations of using this approach to identify and prioritize research questions in the context of a disaster response.

## 1. Introduction

In late 2019, the novel coronavirus SARS-CoV-2 first emerged in Wuhan, China. The virus quickly made its way around the globe and was declared a pandemic by the World Health Organization on March 11, 2020 [1].

Since January 2020, the scientific community’s response to SARS-CoV-2 has been remarkable. Diverse public and private funding agencies made research support available with unprecedented speed. By May 2020, an estimated 23,000 scientific papers addressing myriad topics relevant to COVID-19 or SARS-CoV-2 had been published, with estimates that this number would double approximately every 20 days [2]. Yet, the rapidity in the development of this extensive and unwieldy body of literature raises many concerns about scientific rigor and quality [2]. It also remains unclear if and how responsive the actual spectrum of scientific efforts are in relation to the highest priority scientific gaps and which scientific gaps remain the most pressing. 

### The Need for Coordinated Disaster Research Response (DR2)

Research conducted in parallel to disaster response is critical to learn about the impacts of the event, as well as the effectiveness of response and recovery strategies [3]. Yet, inflexibilities in and unpreparedness of scientific systems—including institutional review boards, funding mechanisms, available protocols, and clear scientific agendas—have thwarted the timely conduct of such research in prior public health emergencies and disasters [3,4,5]. Such needs for vital research have also been recognized by the international community through a call for improved understanding of disaster risks, including dimensions related to vulnerability, capacity, exposure of persons and assets, hazard characteristics, and the environment [6].

In response to the recognized need for research in response to health emergencies, the United States (U.S.) National Institute of Environment Health Sciences (NIEHS), in collaboration with the National Library of Medicine, created the Disaster Research Response (DR2) Program, to promote our ability to conduct time-critical research through publicly accessible data collection tools, pre-event study protocols, networks of trained researchers, multi stakeholder exercises, and integration with emergency management and public health response and recovery planning efforts and frameworks [3,4,5]. NIEHS has also partnered with Japan and Canada to establish similar programs and is working to expand these efforts internationally [7,8].

One of the key components for initiating effective public health emergency research has been the need for quickly identifying and prioritizing research needs in response to specific situations [3,4]. Approaches to this challenge have been variable and include quickly developed workshops convening experts to help evaluate research gaps and greatest areas of importance in response to the Gulf Oil Spill [9] and the Ebola and Zika Outbreaks [10,11]. Following the 2014 Ebola crisis, the World Health Organization similarly established an R&D Blueprint to enable the rapid activation of research and development activities in the context of an epidemic [12].

In early February 2020, the WHO and the Global Research Collaboration for Infectious Disease Preparedness and Response (GLoPID-R) hosted a global research forum where a variety of cross-disciplinary COVID-19 research needs were identified [13]. While the first meeting of the newly formed National Academies of Science, Engineering, and Medicine (NASEM) “Standing Committee on Emerging Infectious Diseases and 21st Century Health Threats” included discussion of COVID-19 research topics as part of its public forum, no broader formal convening was hosted by NASEM in response to COVID-19 for this purpose [14]. Public health measures put in place to slow the spread of SARS-CoV-2 have since precluded in-person meetings ideal for such discussion and deliberation. No such effort has explicitly focused on identifying or prioritizing topics relevant to the environmental and occupational health dimensions of SARS-CoV-2 and COVID-19 research.

## 2. Materials and Methods

In November 2019, the U.S. NIEHS established a DR2 working group as part of its DR2 program to: (1) foster the development of an environmental health researcher “community of practice”; (2) identify environmental health disaster research scientific knowledge gaps and associated research needs in response to specific situations; (3) identify challenges associated with developing and implementing disaster research responses; (4) propose DR2 programmatic strategies and solutions to help address these challenges; and (5) promote collaborations and coordinated research response among the NIEHS community during a disaster.

Prior to the COVID-19 pandemic, the working group had initiated plans, including formation of a new subcommittee, focused on formulating strategies for identifying the environmental health sciences (EHS) research priorities in response to disasters. Recognizing the need to help inform the public of relevant SARS-CoV-2 scientific gaps and needs, the subcommittee quickly transitioned its efforts to focus on the identification and prioritization of EHS questions related to the current pandemic.

In light of travel and gathering restrictions, along with immense personal and professional disruptions brought about by COVID-19, the subcommittee sought a process that could be rapid, remote, and asynchronous. The group decided to employ a modified Delphi method, which deviated from the classic Delphi technique by virtue of it being conducted entirely via email and web-based survey (also known as an e-Delphi or online Delphi) [15]. The Delphi method is a widely used process employed to solicit and refine expert opinion [15,16], where a panel of experts respond to two or more rounds of questionnaires. In the first questionnaire, panelists provide open-ended responses on a certain topic or issue. Analyzed or synthesized responses are then returned to panelists in the form of statements or questions. Panelists rank or rate the statements or questions according to their expert opinion. Panelists receive controlled feedback and are asked to re-rate or re-rank the statements or questions until consensus is achieved, as defined by the study. The Delphi method’s key characteristics include iterative expert input, controlled feedback, participant anonymity, and statistical representation of the group response [15].

**Figure 1:**
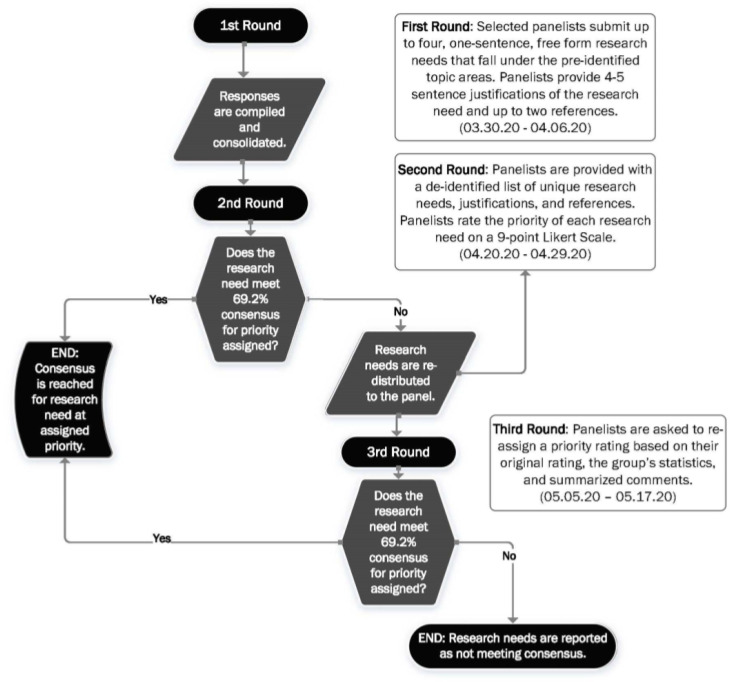
Process Overview.

In our process, panelists were asked to identify research questions and rate their priority through a series of online questionnaires. Following an initial rating, research questions were determined to meet “consensus” at a particular priority category if a certain proportion of panelists assign them a rating within that category. Items that did not reach consensus were redistributed to the panelists, along with feedback including their rating of a particular item and a statistical representation of the overall panel response. They were then asked to repeat the process (i.e., re-rate the remaining items), with the goal of getting closer to a consensus [17]. An overview of our process is provided in Figure 1. As the goal of this activity was to identify high priority environmental and occupational health sciences research needs, and was not research itself, institutional review board approval was not necessary.

### 2.1. Step 1: Identify Diverse Experts

We recruited an expert panel to represent a variety of EHS-related expertise relevant to SARS-CoV-2/COVID-19, including but not limited to: environmental toxicology, environmental microbiology, exposure science, environmental epidemiology, occupational health, environmental public health practice, and environmental health social science. We distributed an email invitation for self-nomination to the panel to members of the NIEHS DR2 Working Group and the Directors of NIEHS Environmental Health Sciences Core Centers [18]. The email encouraged broad distribution to relevant networks. Prospective panelists self-nominated by responding to questions about their expertise and uploading their biosketch or, for practitioners, a four-page resume via a Google Form. We purposefully recruited additional experts from our professional networks to ensure breadth and depth of expertise (e.g., from environmental public health practice). A total of 45 applications were received. The research priorities subcommittee reviewed panelist applications to assess relevance of expertise and to ensure a diverse representation across different environmental and occupational health subdisciplines. This resulted in a total of 28 initial panelists. Applicants received an email notifying them of their selection status, outlining the process and timeline, and were asked to declare any significant financial conflicts of interest and to consent to participation in the process in a Google Form. As this activity was not an NIEHS advisory panel, nor part of any official or unofficial efforts related to NIH funding, current or planned NIEHS grants were not considered conflicts of interest. One selected panelist declined to participate further, and another stopped responding, leaving 26 panelists who participated fully throughout the remainder of the process. Panelists’ self-reported expertise is documented in Table 1.

### 2.2. Step 2: Solicit Research Questions from Panelists

In round 1, panelists were invited to submit up to four, one-sentence, free-form research questions, along with a brief justification and explanation of the associated literature (four to five sentences each) and up to two supporting references through Google Forms. Participants were asked to submit questions reflective of EHS research that should be conducted in the context of the current pandemic to provide public health officials and the general public with additional accurate information about virus transmission, as well as individual and public health measures to limit the spread of disease or reduce its public health consequences. Panelists were provided with the following topics to focus their responses:

- Transmission and routes of exposure;
- Virus survival and infectivity;
- Personal protective equipment (PPE);
- Occupational health impacts and interventions;
- Environmental public health impacts and interventions;
- Environmental health risk communication;
- Cross-cutting areas.

Panelists were provided with instructions about the format for research question submission, along with examples. Instructions to panelists are available in Supplementary File S1, Methods S1. The research priorities subcommittee reviewed, clarified, consolidated, and thematically grouped submitted research questions. A table outlining 61 unique research questions, justifications, and references was developed.

### 2.3. Step 3: Panelists Rate Priority of Research Questions

The developed table with round 1 results was distributed to panelists, along with a REDCap web-administered questionnaire. Study data were collected and managed using REDCap electronic data capture tools [19] hosted at the Institute for Translational Health Sciences. REDCap (Research Electronic Data Capture) is a secure, web-based application designed to support data capture for research studies, providing: (1) an intuitive interface for validated data entry; (2) audit trails for tracking data manipulation and export procedures; (3) automated export procedures for seamless data downloads to common statistical packages; and (4) procedures for importing data from external sources.

In round 2, panelists were asked to rate each question on a nine-point Likert scale (lowest to highest priority). Panelists could also provide explanatory comments, including a justification of their rating. As the research priorities subcommittee felt that some of the research questions submitted may be outside the scope of environmental health sciences, we opted to include an “N/A” option that panelists were instructed to use if they felt the research question was not relevant to environmental health sciences.

We calculated summary statistics and trichotomized responses into “low” (rating = 1, 2, or 3), “medium” (rating = 4, 5, or 6), and “high” (rating = 7, 8, or 9) priority categories at the time of analysis. For each unique research question, we calculated the proportion of respondents who rated at each priority level. We found that the N/A option was not selected by many participants and re-coded N/A responses as “1,” or “lowest priority”. Through comments, we identified that one participant consistently used the N/A option (at the far right of the scale) as a “10”, or highest priority, and re-coded this respondent’s N/A selections as “9”. Each research question where at least 69.2% of panelists’ (*n* = 18) ratings indicated “low priority”, “medium priority”, or “high priority” was determined to meet consensus at that priority rating and eliminated from round 3. While we set a consensus threshold of 70% a priori, we determined that 69.2% was the closest possible proportion given the final number of panelists and that it was not meaningfully different than 70%.

### 2.4. Step 4: Panelists Re-rate Priority of Research Questions, Considering the Group’s Response

Individual reports were developed for each panelist, including the research questions that did not reach consensus in round 2, original justification, individual round 2 ratings, and the “group response”, including mean rating and proportion of responses at each priority level. After consideration of the “group response”, each panelist was asked to re-rate the remaining research questions using a new REDCap web-administered form. The N/A option was eliminated in round 3 given its low use in round 2, and participants were instructed to rate questions that they thought were N/A to environmental health sciences research as lowest priority (i.e., “1”). Summary statistics were again calculated, including the mean and proportion of respondents who indicated “low priority”, “medium priority”, and “high priority”. Research questions that at least 69.2% of panelists rated as “low priority”, “medium priority”, or “high priority” were determined to meet consensus at that priority rating.

## 3. Results

We identified 26 interdisciplinary environmental health science experts to participate in our expert panel. These experts identified 61 unique research questions across several environmental health subdisciplines. Twenty-eight of these research questions were rated as “high priority” by panelists in either round 2 or 3 (Table 2). No research question reached consensus at a “medium” or “low” priority category, potentially indicating reduced discriminatory ability of such a process.

Research questions achieving consensus in the first rating round focused on exposure and transmission variables including timing of infectivity, routes of exposure and their impact on severity, environmental surfaces, particle size in air, and occupational transmission related to work activities. The impact of co-exposure to environmental toxicants and of comorbidities also achieved consensus. The impact on and particular vulnerability of environmental justice communities achieved high priority consensus in the first round, as did understanding the needs of communities with barriers related to education, language, and culture. Prevention-related questions were also prioritized in the first round of rating, including the role of heating, ventilation, and air conditioning (HVAC) systems and public health interventions. The second rating round revealed consensus around similarly themed research questions, but they were often more specific in terms of exposure scenario, prevention strategy, or exposed group. In the weeks between ratings, the media and scientific community brought attention to different issues (e.g., particular essential worker groups and the role of aerosols in disease transmission), which may have also influenced ratings in addition to the group response.

In several cases, the 33 research questions that did not reach consensus could be characterized as very specific in their scope (Supplementary Table S1). These questions received a rating of between 3.73 and 6.46 in round 2, changing to between 3.12 and 6.85 in round 3. This is compared to those questions that did reach consensus in round 2 (ratings between 6.69 and 7.62) and in round 3 (ratings between 6.42 and 7.73). The high rating of some questions that did not reach consensus may be due to the specificity in scope not appreciated by researchers in other areas. These research questions were considered of moderate or high priority by at least 50% of our panel. Our prioritization process should not be construed as defining them as unimportant.

## 4. Discussion

The need for a cohesive community of interdisciplinary disaster scientists “who focus on crises that severely disrupt the environment or threaten human health, and can apply scientific methods in a timely manner to understand how to prevent, mitigate, respond to, or recover from such events” has been recommended [20]. This includes the ongoing development of a community of trained environmental health scientists, in the U.S. and globally, to help provide timely expertise, experience, and applied research in response to health emergencies [5]. The ability for the nascent NIEHS DR2 working group to rapidly transition ongoing collaborative activities to help identify high-priority EHS research questions is a testament to the power of such communities of practice in the context of disaster responses. Yet, there is much work to do to promote capacity and flexibility of such communities to rapidly and cohesively initiate a science response to disasters.

The pace of the COVID-19 science response highlights the imperative to develop hazard-specific research agendas prior to a major disaster response that can be quickly built upon to address situational specificities. While a modified Delphi method offers a remote and relatively quick way to identify research needs, the scientific response has been ongoing and will have vastly outpaced our efforts. As the response to the pandemic is likely to be protracted and occur over multiple waves, we believe these identified research priorities will have significant value in contributing to the identification of scientific needs and opportunities for this ongoing response. Yet, we acknowledge the need for established processes and prepositioned resources to increase the speed of identifying and prioritizing research questions and issues of concern in the future. While event-specific scientific questions can and should be refined and prioritized, initiating processes for research issue identification as a scientific community must occur as a function of scientific preparedness. Formation of a research prioritization process and team whose main purpose would be to manage the administrative process of carrying out similar research issue identification activities in response to future disasters may help streamline the process and provide relevant information to policy-makers, public health officials, and scientific funders earlier in the response.

Finally, the volume of scientific literature that has been produced in the context of the COVID-19 pandemic begs questions about its quality and ability to fully or adequately address scientific needs. A surge of COVID-19 manuscripts published in online preprint servers, preliminary reports of findings released without peer review, has also raised questions about the quality of the work due to accelerated publication [21]. The National Academies of Sciences, Engineering, and Medicine have convened an ad hoc committee to develop and apply methods to review the evidence base for public health preparedness and response practices [22]. However, to the best of our knowledge, no standard accepted practice exists to rapidly review the quality or sufficiency of evidence generated in the context of a disaster. While substantial efforts have been made by scientists to translate rapidly emerging evidence to policy and practice (e.g., Rapid Expert Consultations on the COVID-19 Pandemic by the NASEM Standing Committee on Emerging Infectious Diseases and 21st Century Health Threats) [23], the development of formal processes for rapid evidence typing and grading may help assess whether or not research needs have been met and inform the refinement of future research priorities.

### Limitations

While we chose to select a diverse panel of environmental health expertise, we received feedback that panelists felt unqualified to rate the scientific priority of research questions outside of their particular research domain and requested that a “don’t know” option be considered in future iterations. The extent of the environmental health sciences scientific scope was also apparent in the breadth of ratings. While some participants indicated that certain research areas were outside the scope of environmental health sciences or rated them as low priority, others rated the same questions as high priority. As environmental and occupational health sciences is an inherently interdisciplinary field, future research agenda development may focus on questions specific to particular subdisciplines, with opportunity for cross-disciplinary input, discussion, and synthesis.

While we endeavored to include diverse expertise across the various domains of environmental health sciences in our panel, our closed process did not lend itself to the identification of gaps in the types of expertise included. In addition, while we did not explicitly exclude international participants, we received self-nominations only from panelists employed by organizations located in the U.S. This was likely largely due to our recruitment approach, which focused on U.S. NIEHS grantees and their professional networks. Panelists were not provided with instructions on geographic focus when submitting or rating research priorities. While some of the research priorities identified are clearly applicable (e.g., on routes of transmission) or focused (e.g., on management approaches in areas where WASH access is limited) internationally, the research priorities identified may not reflect global or country-specific priorities outside of the U.S.

We purposefully chose to keep panel participation confidential and anonymous (a key feature of the Delphi technique) throughout the rating process out of concern for unsolicited communication and/or undue influence from individuals or parties with significant financial or other interest in the resultant research priorities. This action was not foolproof; while we asked individual panelists to declare financial conflicts of interest [24], we did not ask them to explicitly refrain from engagement with others when submitting research questions or rating their priority. Moreover, our decision to maintain panelist anonymity precluded panelist interaction, discussion, and deliberation that may have brought clarity or enhanced capacity to achieve consensus, and it inhibited broader scientific, practice, and community contributions to the development of this research agenda.

Evidence is only as useful as it is used. Challenges of evidence-based practice in public health preparedness and response have been well-described and include disparities in perceived information needs between scholars and practitioners [25]. While panel participants included individuals from environmental health practice membership organizations, we did not engage practitioners directly. Future efforts should include more deliberate opportunities for practitioner feedback, along with that of community-based stakeholders, to ensure responsive research that benefits affected communities [26].

## 5. Conclusions

Here, we identified 28 interdisciplinary SARS-CoV-2/COVID-19 environmental health sciences research needs rated as “high priority” by a panel of 26 experts. Prioritizing disaster-specific research questions in the context of a disaster response can help funding organizations prioritize research support, and help researchers focus on projects that matter.

Yet, identifying scientific and operationally relevant information specific to a given disaster is a necessary but insufficient step in public health disaster research response. Future environmental health science preparedness activities should seek to: identify hazard-specific research questions and develop processes for event-specific customization; build organization and workforce capacity to conduct environmental health science disaster research response; and refine processes to assess the quality and sufficiency of environmental health science evidence generated to inform public health and clinical responses in the context of disaster. Given what is sure to be a lengthy response and recovery, alongside a rapidly evolving evidentiary environment, research needs will be inherently dynamic. Processes for refining and updating agendas should be prioritized and resources devoted to their implementation.

## Figures and Tables

**Table 1:** Self-Reported Panelist Expertise.

Expertise	%(*n*) ^a^
Environmental Toxicology	23 (6)
Environmental Microbiology	30.8 (8)
Exposure Sciences	30.8 (8)
Environmental Epidemiology	34.6 (9)
Environmental Public Health Practice	19.4 (7)
Occupational Health	42.3 (11)
Environmental Health Social Sciences	8.7 (4)
Other ^b^	7.7 (2)

^a^ Panelists were able to indicate more than one area of expertise. ^b^ Two panelists reported only “other” expertise (i.e., did not indicate expertise associated with pre-identified categories), including public health emergency preparedness, infectious disease, and molecular toxicology. While other panelists reported expertise in addition to pre-identified categories, they are not reflected in the “other” category above. Additional expertise reported by those panelists included infectious disease, risk communication, environmental justice, children’s health, medicine, and data science.

**Table 2:** Environmental health sciences (EHS) COVID-19 research questions that met consensus threshold as “High Priority”.

**Questions that Reached Consensus in Round 2**	**Ave Rating**
What are the relative contributions of the different disease transmission routes for COVID-19 and how do they vary among exposure scenarios?	7.62
What environmental or occupational exposures might render an individual more susceptible to COVID-19 infection and/or progression of COVID-19-related illness?	7.58
What are the impacts on rates, severity, and outcomes of infection in environmental justice communities where concomitant exposure to contaminants may result in immunocompromised or otherwise increased sensitivity, and poverty and lack of infrastructure can limit ability to implement protective measures?	7.42
Where is SARS-CoV-2 in the environment, and how long does it remain infectious?	7.35
What is the particle size distribution for airborne particles carrying the virus? Does particle size (large droplet vs. small droplet nuclei) make a difference in probability of infection?	7.31
What is the role of HVAC/ventilation in the spread of virus? How can optimized airflow prevent the spread of airborne viruses at high occupancy places?	7.19
What are the real-world risks of infection via fomites on different types of surfaces?	7.08
What is the transmission potential during the initial incubation/asymptomatic phase?	7.04
What are the connections between air pollution, cardiorespiratory diseases, and SARS-CoV-2/COVID-19 severity?	7.04
What are the modes of transmission? Do the modes of transmission affect the severity of symptoms?	6.85
What are the effective public health measures to control the spread of COVID-19 and what has their impact on public health been?	6.85
What patient care activities put healthcare workers at risk of inhaling SARS-CoV-2?	6.81
What can be done to understand the communication needs for communities where education, culture, or language barriers make it difficult to implement effective control strategies to stop the spread of disease and reduce risk?	6.73
How do environmental exposures influence immune defenses and inflammation in the respiratory tract?	6.69
**Questions that Reached Consensus in Round 3**	**Ave Rating**
What is the effect of the environment on transmission?	7.73
What are approaches that can be used to manage the epidemic in contexts where access to water, sanitation, and hygiene (WASH) is minimal (e.g., low-income countries, homeless populations)?	7.69
Droplet transmission of COVID-19 has been demonstrated. To what extent is aerosolization a mode of transmission, including (and especially) in the setting where an aerosolizing procedure is NOT being performed?	7.62
What are potential historical and/or concurrent environmental exposures modulating the severity of COVID-19 in different sub-populations?	7.38
How effective are face masks, and what are the risks of the general public using face masks in reducing community transmissions?	7.31
What are the impacts to those who were employed in minimum wage jobs thought to be extremely low risk, who became the front-line protectors in maintaining supply lines to a society in lockdown?	7.19
What are the co-factors for transmissibility and susceptibility, including role of spread by and to essential services workers?	7.19
How long does the airborne virus maintain infectivity?	7.19
Does air pollution (outdoor and indoor) increase the risk of morbidity and mortality in SARS-CoV-2/COVID-19?	7.08
How will meteorological variables (relative humidity, temperature, rainfall) and seasonality affect the epidemic?	7.00
What are the underlying risk factors for COVID-19?	6.92
What interventions could reduce the lung’s susceptibility to COVID-19 from such environmental or occupational exposures?	6.88
What is the interaction between chemical exposure and COVID-19 morbidity/mortality?	6.81
What are most effective models of risk communication for different segments of the population, given the divergent messages from various forms of media, the changing messages, and what are the expected outcomes of the messages?	6.42

## Supplementary Materials

The following are available online at https://www.mdpi.com/1660-4601/17/18/6842/s1, Supplementary File S1, Methods and Supplementary File S1, Table S1: EHS research questions submitted that did not meet consensus threshold, by Round 3 Average Rating
