# Supplementary material for: Developing an Environmental Health Sciences COVID-19 Research Agenda: Results from the NIEHS Disaster Research Response (DR2) Work Group’s Modified Delphi Method
Source: Int J Environ Res Public Health. 2020 Sep 19;17(18):6842. doi: 10.3390/ijerph17186842 (PMC7557963; doi:10.3390/ijerph17186842)
Supplement: Supplementary file 1 [file ijerph-17-06842-s001.pdf]

File S1: Supplementary Methods: Instructions to Panelists

Round 1:

Dear colleague,

Thank you for your willingness to serve on the NIEHS DR2 Work Group SARS-CoV-2/COVID-19 Environmental Health Research Needs Panel. The panel, composed of a diverse group of environmental and occupational health experts, will identify and prioritize SARS-CoV-2/COVID-19 environmental health research needs. These needs will reflect research that should be conducted in the context of the current pandemic to provide public health officials and the general public with additional accurate information about virus transmission, as well as individual and public health measures to limit the spread of disease or reduce its public health consequences. Specific topics that will be addressed by the panel include:

- Transmission and routes of exposure;
- Virus survival and infectivity;
- Personal protective equipment;
- Occupational health impacts and interventions;
- Environmental public health impacts and interventions;
- Environmental health risk communication;
- Cross-cutting area.

As you may be aware, the National Academy of Medicine has prepared a meeting recap/informal record of issues that describes broad COVID-19 research needs identified by the Standing Committee on Emerging Infectious Diseases and 21st Century Health Threats. Our process endeavors to systematically identify and prioritize *environmental and occupational health specific research needs*. You may wish to review this report to generate ideas about research areas and topics as part of your considerations: https://www.nationalacademies.org/event/03-11-2020/docs/D012DA4393AD7B7552927F6DAB1465FE94C6F336266A

We will be using a modified Delphi method to identify and prioritize research needs across wide ranging environmental and occupational health concerns. The Delphi method is an iterative and systematic way for a group of experts to achieve consensus on a topic. You can read more on the Delphi method here: https://www.rand.org/topics/delphi-method.html

Panelists will be asked to participate in three surveys. Panelists will have 5-7 days to respond to each survey.

In the first survey, panelists will provide up to four environmental health research needs that each fit into any one of the pre-identified topical areas. Research needs should be written as non-specific research questions. In other words, these aren't research questions that require a specific investigator to answer using specific methods in the context of a specific project or grant, but ones that could be answered in a variety of ways through a variety of methods by a variety of investigators. For good examples of the level of question we are looking for, see Boxes 2-5 in the attached National Academies workshop report: "Research Priorities to Inform Public Health and

Medical Practice for Ebola Virus Disease: Workshop in Brief (2014).” Panelists will be asked to provide a 4-5 sentence justification of the research need and current obstacles to answering the research question (if any), including the relevant literature and public health importance, and up to two supporting references.

Responses to this survey will be reviewed by the panel administrators. Redundant submissions will be synthesized and consolidated, and questions may be amended for format in consultation with the submitting panelist.

Panelists will then be provided with a de-identified list of all unique research needs, along with 4-5 sentence justifications and supporting references provided by the submitting panelist in the first survey. In a second survey, panelists will be asked to rate each research need on a three-point Likert scale (low priority, medium priority, high priority). Panelists may also leave comments about the research need or their assessment .

Panel administrators will calculate summary statistics and summarize comments. Any research need where at least 70% of panelists assign the same priority rating will be determined to have reached consensus at that priority assignment.

Research needs that do not meet consensus will be re-distributed to panelists. Panelists will receive an individual report with their own priority assignment rating, the group’s statistics, and summarized comments. Considering the group statistics and summarized comments, panelists will be asked to re-assign a priority rating.

Panel administrators will again calculate summary statistics and summarize comments. Any research need where at least 70% of panelists assign the same priority rating will be determined to have reached consensus at that priority assignment. Research needs that do not ultimately meet consensus will be reported at the priority level assigned by a majority of panelists, and reported as not having met consensus.

We intend to distribute panel findings broadly, including priority assignments and summarized, de-identified comments, and will submit a manuscript documenting these findings to an academic journal. We hope to have findings summarized for distribution and submission by mid-May.

To facilitate your planning, we have provided a timeline of target dates for each round of survey distribution and completion below. Please note that this is our first time using this process, and we may encounter unexpected obstacles. We appreciate your patience, flexibility, and adherence to the timeline as we will endeavor to be as transparent as possible throughout the process.

xxx	Distribution Date	Panelist completion date
Survey 1	Monday, March 30, 2020	Friday, April 3, 2020
Survey 2	Monday, April 13, 2020	Friday, April 17, 2020
Survey 3	Monday, April 27, 2020	Friday, May 1, 2020

As this is our first time using this process to identify disaster-specific research needs, we are eager to learn from our experience. At the end of the process, we will ask you to complete a short evaluation survey and/or participate in a debriefing session.

Please note that this activity is a volunteer effort that is being undertaken by independent investigators who are members of the NIEHS DR2 Work Group. While NIEHS provides infrastructure and support to this group to foster a community of practice to advance our nation's capacity to collectively perform timely research in response to disasters, the group's activities—including this panel—are not formally connected to NIEHS. The panel is not an NIEHS advisory body, and participation on the panel is voluntary.

To promote transparency, we would like to ask all panelists to list any potential financial conflicts of interest related to their participation in this panel. As this is not an NIEHS advisory panel, current or planned NIEHS grants are not considered conflicts of interest. In the event a significant conflict of interest is identified, the panel organizers reserve the right to reconsider your status as panelist.

All data will be reported in aggregate and your responses will not be attributed to you. However, panelists' names, affiliations, subject matter expertise, and self-reported financial conflicts of interests will be included in any reports, presentations, and manuscripts generated from this activity to promote transparency.

You can access the first Delphi survey here: [link]
Please complete your responses no later than COB Friday April 3, 2020.

Please do not hesitate to contact us with any questions, concerns, or technical difficulties. We thank you for your service and look forward to working with you over the next several weeks.

Sincerely,

Nicole Errett, PhD, MSPH, Kimberley Shoaf, DrPH, MPH, and Marilyn Howarth, MD, *on behalf of the NIEHS DR2 Work Group*

Round 2:

Dear colleagues,

Thank you for completing the round 1 survey to identify SARS-CoV-2/COVID-19 environmental health sciences research needs. We have reviewed the submitted research needs and 1) amended for format and clarity and 2) synthesized redundant submissions. In total, we have 61 unique research needs. These research needs, along with their justifications and supporting references, are provided in the attached word document.

We will now ask you to complete a second survey, where you will rate the priority of each research need. The number of the research question in the survey corresponds with the number in the Word document attached, which also includes justifications and references. You can access the second survey here: [link]. **Please complete the survey no later than 9am EDT on Monday April 27th. No late submissions can be accepted to maintain the timelines.**

Please rate the priority for each question on a nine-point Likert scale, where “1” is lowest priority and “9” is highest priority. Your rating should be made considering the importance of conducting the research in the context of the current pandemic to provide public health officials and the general public with additional, accurate information about virus transmission, as well as individual and public health measures to limit the spread of disease or reduce its public health consequences. These learnings may also be applicable to future pandemics.

If you believe the research question is not relevant to environmental health sciences, please select "N/A”. You can optionally include comments/justification about your rating in the text box provided. Synthesized comments may be included in associated reporting, and/or shared with panelists in subsequent rounds to inform their ratings for items that have not yet reached consensus.

Thank you for your continued engagement. Please do not hesitate to reach out with any questions.

Kind regards,

Nicole Errett, PhD, MSPH, Kimberley Shoaf, DrPH, MPH, and Marilyn Howarth, MD, *on behalf of the NIEHS DR2 Work Group*

Round 3:

Dear colleague,

Thank you for completing the round 2 Delphi survey to identify SARS-CoV-2/COVID-19 environmental health sciences research needs. We have summarized the results and have determined 14 research questions have reached "consensus". In other words, at least 69.2% of panelists rated the research question as high priority (7, 8, or 9), medium priority (4, 5, or 6), or low priority (N/A, 1, 2, 3).

We will now ask you to complete a second and final survey, where you will reconsider and re-rate the priority of each research question that has not yet reached consensus. In your re-rating, we ask you to consider the ratings and responses of your peers. As such, we have attached an individualized spreadsheet which includes:

1. The question number *note: the number for each question is the same number in the first round;
2. The original question;
3. Your Round 2 rating;
4. The Round 2 group statistics (average, % panelists that rated at each priority level);
5. Summarized comments from Round 2;
6. The original question justification(s);
7. The original question reference(s).

You can access the second survey here: [link]. **Please complete the survey no later than 5pm EDT on Wednesday, May 13. No late submissions can be accepted to maintain the timelines.**

Again, please rate the priority for each question on a nine-point Likert scale, where "1" is lowest priority and "9" is highest priority. Your rating should be made considering the importance of conducting the research in the context of the current pandemic to provide public health officials and the general public with additional, accurate information about virus transmission, as well as individual and public health measures to limit the spread of disease or reduce its public health consequences. These learnings may also be applicable to future pandemics.

Given the low use of the N/A option in Round 2, we have eliminated it from this round. In our consensus calculations, we grouped N/A ratings with the low priority rating with the rationale that research questions believed "not applicable" to environmental health sciences should be low priority for an environmental health sciences research agenda. As such, if you still believe the research question is not relevant to environmental health sciences, please indicate it as a low priority for an environmental health sciences research agenda.

You can optionally include comments/justification about your rating in the text box provided. Synthesized comments may be included in associated reporting.

Thank you for your continued engagement. Please do not hesitate to reach out with any questions.

Kind regards,

Nicole Errett, PhD, MSPH, Kimberley Shoaf, DrPH, MPH, and Marilyn Howarth, MD, *on behalf of the NIEHS DR2 Work Group*

Table S1. EHS COVID-19 research questions submitted that did not meet consensus threshold, by Round 3 Average Rating.

Question	Round 3				Round 2			
	Average Rating	% High Priority	% Med Priority	% Low Priority	Average Rating	% High Priority	% Med Priority	% Low Priority
What are the outcomes of changes in outdoor air quality that have resulted from COVID-19 containment measures?	6.85	61.54	26.92	11.54	6.38	50	30.77	19.23
What are best practices, including new technology, that could be used to assist with contact tracing?	6.46	57.69	23.08	19.23	6.04	57.69	19.23	23.08
What are the explicate and optimal roles of the environmental health professional in COVID-19 disaster planning, response, and recovery?	6.42	53.85	38.46	7.69	6.46	50	42.31	7.69
How can detailed Quantitative Microbial Risk Assessment (QMRA) models be developed to prospectively inform interventions?	6.38	53.85	34.62	11.54	5.92	50	30.77	19.23
What modes of communication, types of people doing the communication, and types of messages/language used are most effective for different populations, in changing people's behaviors towards greater compliance with recommendations/orders?	6.15	53.85	23.08	23.08	6.31	57.69	23.08	19.23
What is the epidemiology of COVID-19 including using genomic and serologic testing of occupational and non-occupational populations?	6.15	53.85	26.92	19.23	6.27	50	38.46	11.54
What are effective and practical methods of disinfection of the virus on surfaces?	6.08	57.69	23.08	19.23	6.38	65.38	19.23	15.38
Are there alternate hosts/reservoirs for the SARS-CoV-2 virus across the various geographies to which it is spreading and what is their role in transmission?	6.08	53.85	34.62	11.54	6	50	34.62	15.38
What material properties limit the survival and infectivity of SARS-CoV-2?	6.04	30.77	61.54	7.69	6	42.31	46.15	11.54
What are the unintended health effects of the “stay-at-home” requirements (e.g. exposure to indoor environments including indoor air, lead, pesticides for longer periods of time)?	6	42.31	42.31	15.38	5.46	34.62	38.46	26.92
What are reasonable steps that individuals can do at home to protect themselves from COVID-19 and how can we message that information?	5.96	38.46	46.15	15.38	5.5	46.15	26.92	26.92
What are the data on worker fatigue, willingness to work, exposure to COVID-19, post-traumatic stress disorder (PTSD), etc., given the pervasive PPE issues with the pandemic, as well as the extensive press coverage and multiple media (popular, social) reports related to PPE use and abuse, and the changing guidance from Centers for Disease Control and Prevention (CDC), WHO, etc.?	5.92	50	38.46	11.54	5.69	42.31	42.31	15.38
What are the long-term health risks, especially on cardiovascular, respiratory, and mental health, of COVID-19 infection in various vulnerable populations?	5.88	57.69	15.38	26.92	6.15	61.54	23.08	15.38

Do changes in cleaning products/practices change the environment or affect worker (or public) health?	5.85	42.31	42.31	15.38	6.27	57.69	30.77	11.54
What are the short- and long-term environmental impacts of the increased medical waste from COVID-19?	5.81	34.62	50	15.38	5.42	38.46	42.31	19.23
How has the decentralized nature of risk communication across federal, state, and local preparedness tiers influenced uptake of evidence-based information about COVID-19 transmission and prevention and health behaviors?	5.77	42.31	30.77	26.92	5.58	34.62	46.15	19.23
What research is needed to design reusable N95 respirators that can be cleaned/disinfected multiple times in order to avoid the huge delays, mask hoarding, and environmental pollution?	5.54	38.46	42.31	19.23	6	53.85	34.62	11.54
What are the triggers or assessments that should initiate interventions? What type of routine monitoring should be occurring to identify this in the future?	5.42	34.62	42.31	23.08	5.88	53.85	23.08	23.08
What is the optimal method to decontaminate the N95 respirator that will also ensure access to the process?	5.23	23.08	61.54	15.38	6.04	50	34.62	15.38
What are post-pandemic facility re-occupancy criteria, including premise plumbing?	5.19	23.08	57.69	19.23	5.31	34.62	42.31	23.08
How have online social interactions mediated the perceived credibility of expert and peer messages about COVID-19 transmission and prevention, including both the provision of new evidence-based information and the correction of misinformation?	5	26.92	46.15	26.92	5.54	38.46	42.31	19.23
What are the immune-mediated responses and pathology to SARS-CoV-2 infection?	5	34.62	26.92	38.46	5.38	42.31	38.46	19.23
Have local (and state) emergency policies that affect housing quality and stability affected health?	5	23.08	61.54	15.38	5	26.92	46.15	26.92
What strategies can be used to minimize or eliminate shortages of personal protective equipment during pandemics?	4.92	30.77	38.46	30.77	5.81	42.31	34.62	23.08
What design changes can be made to identify or develop PPE that is usable and effective in a range of temperatures or that is specifically designed to meet the needs of one temperature extreme, such as high temperature climates?	4.88	23.08	46.15	30.77	5.31	34.62	46.15	19.23
How are health organizations at different preparedness tiers amplifying and/or creating COVID-19 information online, and how is that changing over the course of the pandemic?	4.85	19.23	57.69	23.08	4.81	23.08	46.15	30.77
What steps are being taken to support rapid studies to support Food and Drug Administration (FDA) Emergency Use Authorization (EUA) on PPE reuse and to understand the risks associated with reuse?	4.31	11.54	61.54	26.92	4.88	19.23	57.69	23.08

How does SARS-CoV-2/COVID-19 influence and infect non-respiratory tract tissues, including the brain and nervous system?	4.31	23.08	26.92	50	4.88	34.62	38.46	26.92
What airway innate immune mechanisms affect SARS-CoV-2 infection?	4.15	23.08	26.92	50	5.27	38.46	38.46	23.08
What is the potential for transmission from other environmental sources (e.g., drinking water, swimming pools, lakes)?	3.81	7.69	46.15	46.15	4.88	34.62	30.77	34.62
What research should be conducted in different waves of the pandemic?	3.69	19.23	23.08	57.69	3.73	15.38	34.62	50
Has SARS-CoV-2 generated the mutations that altered the virulence and infectivity during the human transmission?	3.46	11.54	26.92	61.54	4.19	19.23	38.46	42.31
Does facial hair (e.g., moustache, full beard) significantly reduce the effectiveness of half-mask respiratory protection (because of an inadequate seal—a problem with regards to occupational exposures to small particles, unknown as it pertains to COVID-19 associated aerosols).	3.12	3.85	34.62	61.54	4.5	19.23	42.31	38.46
